# Fermentation Parameters, Amino Acids Profile, Biogenic Amines Formation, and Bacterial Community of Ensiled Stylo Treated with Formic Acid or Sugar

**DOI:** 10.3390/ani14162397

**Published:** 2024-08-18

**Authors:** Kai Mao, Marcia Franco, Yi Xu, Huan Chai, Jian Wang, Shuai Huang, Zhiyong Wang, Wenjuan Xun, Zuoxiang Liang, Zhu Yu, Musen Wang

**Affiliations:** 1 School of Tropical Agriculture and Forestry, Hainan University, Haikou 570228, China; maokai@hainanu.edu.cn (K.M.); xuyijoker@163.com (Y.X.); chaihuan@hainanu.edu.cn (H.C.); wangjian901@163.com (J.W.); huangshuai@hainanu.edu.cn (S.H.); wangzhiyong@hainanu.edu.cn (Z.W.); xunwenjuan991@163.com (W.X.); 2 Natural Resources Institute Finland (Luke), Tietotie 4, FI-31600 Jokioinen, Finland; marcia.franco@luke.fi; 3 Department of Animal Science, University of Minnesota, Saint Paul, MN 55108, USA; zliang@umn.edu; 4 College of Grassland Science and Technology, China Agricultural University, Beijing 100193, China; yuzhu02059@163.com

**Keywords:** *Stylosanthes guianensis*, ensiling, putrescine, cadaverine, tyramine, microflora

## Abstract

**Simple Summary:**

Stylo silage is widely used as a protein source for ruminants in tropical and subtropical regions. High levels of biogenic amines in stylo silage are harmful to the health of ruminant animals. The purpose of this work was to evaluate the effect of formic acid and sugar on biogenic amines and amino acids concentrations and bacterial composition and fermentation profile of stylo silage. The results showed that putrescine, cadaverine, and tyramine became predominant in the control silage, and applying formic acid and sugar significantly decreased putrescine, cadaverine, tyramine, and total biogenic amine concentrations. *Clostridium pabulibutyricum*, *Weissella cibaria* and *W. paramesenteroides* were the predominant bacteria in the control silage, and the application of both additives remarkably lowered their relative abundance. Correlation analysis showed that *C. pabulibutyricum*, *W. cibaria* and *W. paramesenteroides* were positively related to putrescine, cadaverine, and tyramine formation. The application of formic acid or sugar significantly reduced the undesirable bacterial population and improved the fermentation and hygienic quality of the stylo silage.

**Abstract:**

Substantial proteolysis occurs and free amino acids can be degraded to biogenic amines by decarboxylation during stylo (*Stylosanthes guianensis*) ensiling. High biogenic amine concentrations in silage are harmful to the health of ruminant animals. The purposes of this work were to (1) analyze the biogenic amines and amino acids concentrations, bacterial composition, and fermentation profile of spontaneously fermented stylo silage, (2) explore the effect of formic acid or sugar additive on these silage parameters, and (3) further reveal the correlations between silage amines and fermentation parameters, amino acids, and bacteria. Freshly chopped stylo was treated with distilled water (control), formic acid (4 mL/kg), and sugar (20 g/kg) and fermented for 28 days. The results indicated that putrescine (321 mg/kg dry matter), cadaverine (384 mg/kg dry matter), and tyramine (127 mg/kg dry matter) rapidly increased in concentration and become predominant in the control silage after 28 days of fermentation. Applying formic acid and sugar at ensiling, especially the acidifier, significantly decreased putrescine, cadaverine, tyramine, and total biogenic amine concentrations compared with the control treatment (*p* < 0.0001). *Clostridium pabulibutyricum*, *Weissella cibaria* and *W. paramesenteroides* were the predominant bacteria in the control silage, and the application of both additives remarkably lowered their relative abundance in comparison with the control treatment (*p* < 0.001). Correlation analysis showed that putrescine, cadaverine, and tyramine were positively related to pH, butyric acid, non-protein nitrogen, and ammonia nitrogen (*p* < 0.01). These amines also had significant correlations with *C. pabulibutyricum*, *W. cibaria* and *W. paramesenteroides* (*p* < 0.001). Putrescine, cadaverine, and tyramine were the main biogenic amines and *C. pabulibutyricum* was the predominant undesirable bacterium in naturally fermented stylo silage. *C. pabulibutyricum*, *W. cibaria* and *W. paramesenteroides* were positively related to putrescine, cadaverine, and tyramine formation. The application of formic acid or sugar significantly reduced the undesirable bacterial population and improved the fermentation and hygienic quality of the stylo silage. These findings lay the foundation for further elucidating the microbial mechanism underlying the main biogenic amine formation during fermentation of stylo silage.

## 1. Introduction

Stylo (*Stylosanthes guianensis*) is an important legume forage in tropical and subtropical regions due to strong adaptation to low-fertility soils and high quality and yield [1,2,3]. Under high temperature and humidity conditions, ensiling is a common way of conserving stylo. However, a significant proportion of protein undergoes proteolysis during stylo ensiling [4], and free amino acids can be further degraded to ammonia and biogenic amines by deamination and decarboxylation [5], respectively. High biogenic amine concentrations in silage decrease the palatability and feed intake by ruminants, impair rumen mucosa, induce immunological diseases, and threaten the safety of animal products [6,7].

Biogenic amines are low-molecular-weight compounds containing N [8] and classified into aliphatic amines (putrescine, spermidine, cadaverine and spermine), aromatic amines (phenylethylamine and tyramine) and heterocyclic amines (histamine and tryptamine) according to their chemical structure [9]. The basic conditions for biogenic amine formation consist of plentiful free amino acid availability [10], microorganisms with amino acid decarboxylase activity [11,12], and a suitable environment for microorganism growth and decarboxylase activity [12]. In ensiled forages and fermented feeds, the precursors for biogenic amine synthesis are ornithine (putrescine), lysine (cadaverine), tyrosine (tyramine), phenylalanine (phenylethylamine), tryptophan (tryptamine), and histidine (histamine) [9,13]. Biogenic amines are produced by various microorganisms, consisting of mainly bacteria and some fungi. Santos [9] reported that *Enterobacter cloacae* produced putrescine and *Klebsiella pneumonia* produced cadaverine and histamine. Similarly, Li et al. [14] found that putrescine was formed mainly by *Enterobacter cloacae*, *Escherichia coli* and *Citrobacter* sp. and *Escherichia coli* and *Klebsiella oxytoca* were the key bacteria for cadaverine and tyramine production in alfalfa silage.

So far, the research on stylo silage has mainly focused on fermentation profile, proteolysis, bacterial community and in vitro rumen fermentation. However, biogenic amine profile analysis remains unexplored. In addition, it is reported that formic acid and sugar significantly suppressed the biogenic amines formation of perennial ryegrass and alfalfa silages [14,15], respectively. Therefore, the purposes of this work were to (1) analyze the biogenic amines and amino acids concentrations, bacterial composition, and fermentation profile of spontaneously fermented stylo silage, (2) explore the effect of formic acid or sugar on these silage parameters, and (3) further reveal the correlations among silage amines and fermentation parameters, amino acids, and bacteria.

## 2. Materials and Methods

### 2.1. Silage Preparation

The stylo (Reyan no. 2) forage was grown in the experimental fields of Hainan University (latitude 20°5′ N, longitude 110°32′ E), where there were three plots (about 20 m^2^ each) growing stylo at the late budding period. The forage for the first cut in each plot was randomly sampled, manually harvested at a stubble height of 10 cm and chopped into approximately 2–3 cm pieces by a forage chopper on 2 June 2022. Freshly chopped forage was collected in a pile. Three piles in total were thoroughly mixed, divided into 12 small stacks, and randomly subjected to three treatments: distilled water (control, CK), formic acid (4 mL/kg, FA; CH_2_O_2_, analytic reagent, Sinopharm Group Chemical Reagent Co., Ltd., Beijing, China), and sugarcane-derived sugar (20 g/kg, SU; C_12_H_12_O_11_, analytic reagent, Sinopharm Group Chemical Reagent Co., Ltd., Beijing, China). A 30 mL diluted FA solution was prepared by dissolving 4 mL of FA into distilled water. Similarly, 30 mL of SU solution was prepared by dissolving 20 g of SU into distilled water. The prepared additives were individually sprayed on the 1 kg of chopped forage with a 50 mL spray bottle. The CK treatment was treated with the same volume of distilled water. Each treatment was prepared with four replicates. A total of 12 plastic bag silos (25 cm × 35 cm, 250 g/silo) were fermented for 28 days at room temperature (about 25 °C). Fresh forage was packed into four silo bags and vacuumized and immediately stored at −20 °C for further analysis.

### 2.2. Fermentation Parameters Analysis

At silo opening, 20 g of fresh forage or silage and 180 mL of distilled water were juiced for 1 min and filtered with three layers of sterile gauze. A portion of the filtrate was employed to determine the pH value by a pH meter (PHS-3C, INESA, Shanghai, China), and was subsequently subject to centrifugation (8000× *g*) for 10 min at 4 °C. The centrifuged filtrate was used to determine lactic acid (LA), acetic acid (AA), propionic acid (PA) and butyric acid (BA) by high performance liquid chromatograph (Shimadzu-10A, Kyoto, Japan) according to Wang et al. [16]. The other portion of the filtrate (40 mL) and 10 mL of 20% trichloroacetic acid were thoroughly mixed, stored at 4 °C overnight to precipitate protein and centrifuged (10,000× *g*) for 15 min at 4 °C. The supernatant was employed to analyze ammonia nitrogen (ammonia-N) and free amino acid nitrogen (FAA-N) concentrations via the colorimetric method according to Broderick and Kang [17]. One hundred grams of sample was used to determine dry matter (DM) content after 72 h of lyophilization, ground through a 1 mm sieve with a laboratory knife mill and stored at 4 °C for chemical analysis. Water-soluble carbohydrates (WSC) were analyzed in accordance with Murphy [18]. Total nitrogen (TN) and non-protein nitrogen (NPN) were determined according to the AOAC [19] and Sniffen et al. [20], respectively. Crude protein (CP) determination arose from TN × 6.25. Peptide nitrogen (peptide-N) was estimated through the difference between NPN and the sum of ammonia-N and FAA-N. According to Mertens [21], the AOAC Official First Action method without a heat-stable amylase was used to determine neutral detergent fiber (NDF). Acid detergent fiber (ADF) was determined by the method as described by the AOAC [22]. The NDF and ADF data are shown inclusive of residual ash. Depending on organic acids (percentage of fresh weight) and ammonia-N (percentage of TN) indices, the V-score evaluation system (Table S1) was used to evaluate silage fermentation quality [23]. Based on the V-score, silage fermentation quality was divided into three groups, consisting of excellent level (>80), acceptable level (60–80), and poor level (<60).

### 2.3. Amino Acids Analysis

Determination of 24 free amino acids was performed by liquid chromatography–mass spectrometry (LC-MS, Fuzhou Bereys Biotechnology Co., Ltd., Fuzhou, China). Freeze-dried powder of about 50 mg was extracted with 0.6 mL of 0.1 M hydrochloric acid for 1 h with gentle agitation in a shaker and centrifuged (12,000× *g*) for 10 min. After a series of dilutions, the supernatant was filtered through a 0.22 μm-pore membrane filter. A mixture of 10 μL of the filtrate, 70 μL of AccQ·Tag Ultra Borate buffer (Waters Corporation, MA, USA) and 20 μL of AccQ·Tag (Waters Corporation, MA, USA) was kept for 1 min at room temperature, heated for 10 min at 55 °C and cooled for analysis by ultrahigh-performance liquid chromatography (Vanquish, Thermo Fisher Scientific, MA, USA) and mass spectrometry (Q Exactive, Thermo Fisher Scientific, MA, USA). The whole analysis process was undertaken in an autosampler at 4 °C. The parameters of liquid chromatography were as follows: column, Waters Acquity UPLC BEH C18 (50 mm × 2.1 mm × 1.7 μm); column temperature, 55 °C; flow rate, 0.5 mL/min; injection volume, 1 μL; mobile phase consisting of formic acid/ultrapure water (1/999, *v*/*v*; A) and formic acid/chromatographic grade acetonitrile (1/999, *v*/*v*; B) and gradient program consisting of 95:5 (*v*/*v*) at 0 min, 90:10 at 5.5 min, 75:25 at 7.5 min, 40:60 at 8 min, 95:5 at 8.5 min and 95:5 at 13 min. The amino acid content of sample was calculated as follows: amino acid concentration of the sample (mg/kg DM) = (C × V × F × M_w_)/M; C, sample concentration (nmol/mL); V, constant final sample volume (mL); F, sample dilution ratio; M_w_, amino acid molecular weight; M, sample weighing mass (mg). Total amino acid concentration was the sum of the amount of the individual amino acids.

The external standard method was employed in the analysis, and the 24 amino acid standard substances were from Sigma-Aldrich Corporation (Saint Louis, MI, USA). A stock solution (100 μmol/mL) of each amino acid was prepared using 0.1 M hydrochloric acid, and 0.5, 1, 5, 10, 50, 100, 150, 200 and 300 nmol/mL solutions containing an amino acids mixture were prepared. The detection levels for asparagine, lysine, cystine and the remaining amino acids were 1 and 0.5 nmol/mL, respectively.

### 2.4. Biogenic Amines Analysis

Eight biogenic amines were determined by LC-MS (Fuzhou Bereys Biotechnology Co., Ltd., Fuzhou, China), similar to amino acids in the analysis procedure. Freeze-dried powder of about 50 mg was extracted with 0.5 mL of 0.1 M hydrochloric acid for 1 h with gentle agitation on a shaker and centrifuged (12,000× *g*) for 10 min. The pretreatment steps and the parameters of LC-MS were in accordance with the aforementioned amino acids analysis. The biogenic amine concentration of sample was calculated as follows: sample biogenic amine content (mg/kg DM) = (C × V × F × M_w_)/M; C, sample concentration (nmol/mL); V, final constant volume of sample (mL); F, sample dilution ratio; M_w_, biogenic amine molecular weight; M, weighed mass of sample (mg). Total biogenic amine concentration arose from the sum of the amount of the individual biogenic amines.

The external standard method was employed in the analysis and eight biogenic amine standard substances were purchased from Sigma-Aldrich Corporation (Saint Louis, MI, USA). The stock solution (100 μmol/mL) of each biogenic amine was prepared using 0.1 M hydrochloric acid, and 1, 5, 10, 50, 100, 150, 200, 250, 300 and 500 nmol/mL of solution containing a mixture of biogenic amines were prepared. The detection levels for putrescine and the remaining biogenic amines were 1 and 0.5 nmol/mL, respectively.

### 2.5. Bacterial Community Analysis

The pellet of silage sample was prepared according to Wang et al. [24]. DNA extraction, amplicon sequencing and sequence and bioinformatics analysis were performed at Shanghai Personal Biotechnology Co., Ltd. (Shanghai, China). Total DNA was extracted by a soil MagBeads FastDNA Kit (MP Biomedicals, CA, USA). The quantity and quality of extracted DNA were determined by a NanoDrop NC2000 spectrophotometer and agarose gel electrophoresis, respectively. PCR amplification of the nearly full-length bacterial 16S rRNA genes was performed using the forward primer 27F (5′-AGAGTTTGATCMTGGCTCAG-3′) and the reverse primer 1492R (5′-ACCTTGTTACGACTT-3′). A set of 10-nucleotide barcodes were incorporated into each primer. The PCR components contained 5 µL of Q5 reaction buffer (5×), 5 µL of Q5 high-fidelity GC buffer (5×), 0.25 µL of Q5 high-fidelity DNA polymerase (5 U/µL), 2 µL of dNTPs (2.5 mM), 1 µL of each forward and reverse primer (10 µM), 2 µL of DNA template and 8.75 µL of ddH_2_O. The procedure consisted of at 98 °C for 2 min, 25 cycles of denaturation at 98 °C for 30 s, annealing at 56 °C for 30 s and extension at 72 °C for 45 s, with a final extension of 10 min at 72 °C. Sequence data analyses were done mainly by QIIME2 and R pakage (v3.6.0). Alpha diversity indices (Chao1, observed species, Shannon and Simpson parameters) were calculated in QIIME2 and visualized as box plots. Hierarchical clustering (beta diversity) analysis was performed to investigate the structural variation of bacterial community across samples according to Ramette [25]. Taxonomy composition and abundance were visualized using MEGAN [26] and GraPhlAn [27].

### 2.6. Statistical Analysis

One-way analysis of variance was performed using the Statistical Package for Social Science (SPSS 20, Inc., Chicago, IL, USA). The applied model was A_i_ = μ + B_i_ + C_i_, where A_i_ is the observation, μ is the mean, B_i_ is the treatment influence, and C_i_ is the residual error. Duncan’s test was employed to contrast the means, and significance was declared at *p* < 0.05. The correlation analysis between silage amines and fermentation parameters, amino acids, and bacteria was performed by R software (v3.6.0).

## 3. Results

### 3.1. Chemical Characteristics of Stylo Prior to Ensiling

As shown in Table 1, the DM, WSC and CP contents of stylo prior to ensiling were 248 g/kg fresh weight, 21.5 g/kg DM and 132 g/kg DM, respectively, and the NPN level was 125 g/kg TN. The contents of NDF and ADF were 595 g/kg DM and 445 g/kg DM, accordingly.

### 3.2. Fermentation Parameters of Stylo Silage

As shown in Table 2, the CK silage was poorly fermented, indicated by low LA content (12.8 g/kg DM) and high levels of pH (4.87), BA (16.1 g/kg DM) and ammonia-N (80.4 g/kg TN), after 28 days of conservation. As a result, its fermentation evaluation score was 59.5. In contrast, the fermentation scores of the FA and SU silages were 90.0 and 99.3, respectively. The NPN concentration in the CK silage was 624 g/kg TN and the application of FA and SU, especially FA, significantly decreased NPN concentration compared with the CK treatment (*p* < 0.0001).

### 3.3. Amino Acids Profile of Fresh and Ensiled Stylo

The fresh stylo prior to ensiling was rich in asparagine (2226 mg/kg DM), glutamine (1122 mg/kg DM), arginine (641 mg/kg DM), leucine (499 mg/kg DM) and alanine (491 mg/kg DM) (Table 3). After 28 days of ensiling, the majority of the 24 amino acids in the CK silage to different extents had increased in concentration, except for serine, asparagine, glutamine, arginine and cystine. Applying FA remarkably decreased the contents of methionine, tryptophan, valine, phenylalanine, leucine, isoleucine, threonine, histidine, glycine, aspartic acid, glutamic acid, alanine, proline, tyrosine, ornithine, γ-aminobutyric acid, D-α-aminobutyric acid and 4-hydroxy-L-proline in comparison with CK and SU treatments (*p* < 0.01). Lysine, threonine, serine and ornithine levels were higher in the SU treatment than the CK and FA treatments (*p* < 0.01). Total amino acid content of stylo prior to ensiling was 8530 mg/kg DM, and its content in the CK, FA and SU treatments was 21,102 mg/kg DM, 8951 mg/kg DM and 17,452 mg/kg DM, respectively, after 28 days of fermentation.

### 3.4. Biogenic Amines Formation of Fresh and Ensiled Stylo

The biogenic amine concentrations of fresh and ensiled stylo are shown in Table 4. Putrescine (125 mg/kg DM) was the main amine in fresh stylo, followed by spermidine (39.4 mg/kg DM), spermine (14.0 mg/kg DM), cadaverine (9.26 mg/kg DM) and tyramine (8.39 mg/kg DM). After 28 days of fermentation, putrescine (321 mg/kg DM), cadaverine (384 mg/kg DM) and tyramine (127 mg/kg DM) rapidly increased in concentration and became predominant in the CK silage. The application of FA or SU significantly decreased concentrations of putrescine, cadaverine and tyramine (*p* < 0.001). Total biogenic amine concentration of fresh stylo prior to ensiling was 199 mg/kg DM and its content in silage had increased after 28 days of fermentation. The highest concentration of total biogenic amines was found in the CK treatment (866 mg/kg DM), followed by the SU treatment (331 mg/kg DM), and finally the one with the lowest concentration among the silage was the FA treatment (225 mg/kg DM).

### 3.5. Bacterial Community of Stylo Silage

**Figure 1:**
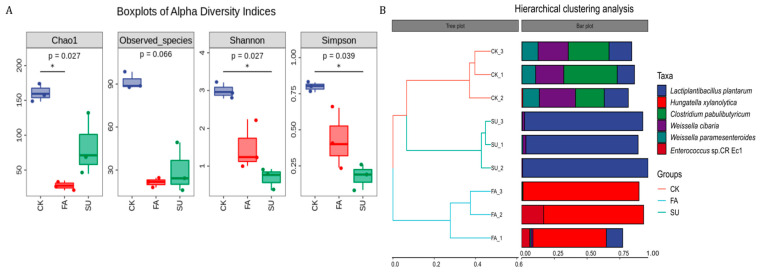
Alpha diversity indices (**A**) and clustering analysis (**B**) of bacterial community in stylo silage (n = 3). CK = control; FA = formic acid; SU = sugar; “*” indicates a significant difference (*p* < 0.05).

The alpha diversity indices and clustering analysis of bacterial community in stylo silage are presented in Figure 1. The Chao1 index value in the CK silage was higher than the FA treatment (*p* < 0.05) and Shannon and Simpson index readings in the SU treatment were lower compared to the CK treatment (*p* < 0.05, Figure 1A). Figure 1B shows the clustering analysis of the bacterial community in silage, which indicated that nine silage samples were clearly divided into three different clusters.

As shown in Table 5, the predominant bacteria in the CK silage were *Clostridium pabulibutyricum* (31.2%), *Weissella cibaria* (24.1%), *W. paramesenteroides* (11.8%) and *Lactiplantibacillus plantarum* (16.3%). The application of FA or SU significantly reduced *C. pabulibutyricum*, *W. cibaria* and *W. paramesenteroides* relative abundance (*p* < 0.001). *L. plantarum* (90.6%) and *Hungatella xylanolytica* (73.6%) were the only dominant bacteria in the FA and SU silages, respectively.

### 3.6. Correlations between Biogenic Amines and Fermentation Parameters, Amino Acids, and Bacteria

**Figure 2:**
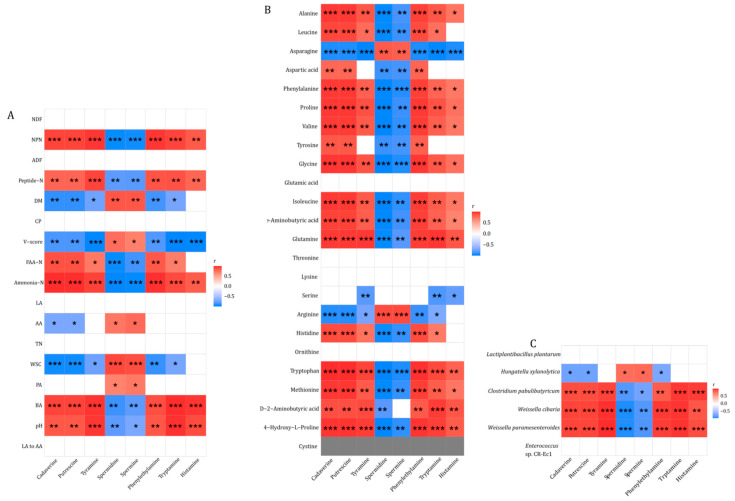
Correlations between biogenic amines and fermentation parameters (**A**), amino acids (**B**) and bacteria (**C**) (n = 3). LA = lactic acid; AA = acetic acid; PA = propionic acid; BA = butyric acid; DM = dry matter; Ammonia-N = ammonia nitrogen; TN = total nitrogen; WSC = water-soluble carbohydrates; NDF = neutral detergent fiber; ADF = acid detergent fiber; CP = crude protein; NPN = non-protein nitrogen; Peptide-N = peptide nitrogen; FAA-N = free amino acid nitrogen; “*,” “**” and “***” indicate “*p* < 0.05,” “*p* < 0.01” and “*p* < 0.001,” respectively.

The correlations between silage amines and fermentation parameters, amino acids and bacteria are shown in Figure 2. Putrescine, cadaverine and tyramine were positively related to NPN, Peptide-N, FAA-N, Ammonia-N, BA and pH (*p* < 0.05) and negatively related to DM, V-score and WSC (*p* < 0.05) (Figure 2A). Phenylethylamine and tryptamine had significant correlations with phenylalanine and tryptophan (*p* < 0.001, Figure 2B), respectively. Arginine was positively correlated with spermidine and spermine (*p* < 0.001) and negatively correlated with putrescine (*p* < 0.001). No significant correlation (*p* > 0.05) was observed between cadaverine and lysine, putrescine and ornithine, or tyramine and tyrosine. Similarly, histamine did not have a significant correlation with histidine (*p* > 0.05). Putrescine, cadaverine, tyramine, phenylethylamine, tryptamine and histamine were positively related to *C. pabulibutyricum*, *W. cibaria* and *W. paramesenteroides* (*p* < 0.01, Figure 2C).

## 4. Discussion

In the present work, the CK silage was poorly fermented, indicated by high pH, ammonia-N, and BA levels and a low LA concentration, due to low DM and WSC concentrations, and subsequently an extensive proteolysis occurred, which was in agreement with the results of Zhang et al. [3], He et al. [4], Wang et al. [28] and Chen et al. [29]. The WSC content in FA treatment (23.5 g/kg DM) was significantly higher than SU treatment (14.3 g/kg DM). This finding could be due to the fact that FA, a fermentation inhibitor, can inhibit most microorganisms, including many lactic acid bacteria, by direct acidification at ensiling [15], and subsequently more residual WSCs are reserved and less LA was produced in FA treatment. In contrast, SU is a fermentation promoter and can promote lactic acid bacteria activity in the early stage of fermentation [14], and as a result, most WSCs were converted into organic acids mainly consisting of LA, thereby resulting in an extensive decrease in pH and WSC levels in SU treatment. The AA content (43.8 g/kg DM) in the FA silage was greatly higher than the CK and SU treatments, which disagrees with the results of Van Os et al. [15] and Song et al. [30]. One possible explanation is that the low-pH environment induced by FA promoted activity of some acetic acid bacteria at the early stage of fermentation in stylo silage [31]. A great deal of BA was produced in the CK silage, possibly because some *Clostridium* sp. exceeded lactic acid bacteria and converted part of LA, formed by lactic acid bacteria in the early stage of fermentation, into BA at the middle and late stages of fermentation [31].

Poor fermentation in silage was generally accompanied by an extensive proteolysis [16,32,33], and peptides during proteolysis can be further degraded to amino acids. Of the 24 free amino acids tested, lysine, arginine, ornithine, tyrosine, tryptophan, phenylalanine and histidine concentrations rapidly increased and arginine concentration extensively decreased in spontaneously fermented stylo silage after 28 days of ensiling. According to Guo et al. [34], there were decreases in threonine, serine, glutamic acid, lysine, histidine and arginine, and increases in aspartic acid, branched chain amino acids valine, isoleucine and leucine in the untreated alfalfa silage compared with the fresh alfalfa, which was similar to the results by Fairbairn et al. [35] and Winters et al. [36] and did not agree with our results to a large extent. The variation across different phenomena can be attributed to a series of factors such as forage species and wilting. In addition, the application of FA increased serine and arginine levels in the present work, which was in agreement with Guo et al. [34]. However, we observed that FA decreased contents of valine, phenylalanine, threonine, histidine, aspartic acid, glutamic acid, tyrosine and ornithine, and this difference between our findings and those of Guo et al. [34] needs to be further explored.

Free amino acid can be decarboxylated into biogenic amine and biogenic amines in silage consists of endogenous amines and exogenous amines. According to the data of our present work, the predominant amine in fresh forage was putrescine, which agreed with the results of Van Os et al. [15], Nishino et al. [37], Li et al. [14] and Jia et al. [38,39]. After 28 days of fermentation, a great deal of putrescine, cadaverine and tyramine were formed in the CK treatment. Similarly, Steidlová and Kalač [40,41], Jia et al. [38] and Li et al. [14] reported that high putrescine, cadaverine and tyramine concentrations were produced in naturally fermented alfalfa, oat and grass silages. The application of SU and FA decreased their concentrations in alfalfa and grass silages [14,15,41], which was confirmed by our results. The biosynthesis of biogenic amines in silage mainly consists of amino acid decarboxylation pathway [9] and also occurs via amination and transamination of aldehydes and ketones in some cases [42]. The precursors for putrescine, cadaverine and tyramine formation are ornithine, lysine and tyrosine [6], respectively. Generally, the larger the precursor amino acid concentration, the higher the corresponding biogenic amine level. Interestingly, our results did not reveal a significant correlation between ornithine and putrescine, lysine and cadaverine, or tyrosine and tyramine. The amino acid concentrations of the forage prior to ensiling affected the amino acid concentrations in silage to some extent, with total amino acid concentrations being generally higher in the silage than in the material prior to ensiling. A possible explanation is that amino acid biosynthesis (such as peptide hydrolysis) and degradation (such as deamination or decarboxylation) simultaneously occurs during stylo ensiling, and the remaining amino acid concentration will generally be higher if its biosynthesis rate is greater than its degradation rate. One amino acid can also be converted into another. For instance, arginine can be converted into ornithine by arginase [43]. Furthermore, ornithine, lysine and tyrosine might have been partly decarboxylated into putrescine, cadaverine and tyramine by ornithine decarboxylase, lysine decarboxylase and tyrosine decarboxylase, respectively, during fermentation of silage [14].

Free amino acids in silage can be decarboxylated into biogenic amines via amino acid decarboxylases of microorganisms [9]. Bacteria often play an important role in biogenic amine formation [44]. It has been proven that lactic acid bacteria (*Weissella*, *Pediococcus*, *Streptococcus*, *Leuconostoc* and *Lactobacillus*), *Clostridium* and *Enterobacteriaceae* (*Citrobacter*, *Escherichia*, *Klebsiella* and *Salmonella*) are important biogenic amine producers [8,9,14,45]. Similarly, our work indicated that *W. cibaria* and *W. paramesenteroides* had positive correlations with putrescine, cadaverine and tyramine. These two lactic acid bacteria often grow and proliferate in weak acidic environments and secrete amino acid decarboxylases, whose activity is optimal within the pH range of 4.0–5.5 [46]. Our findings confirmed this, possibly because the amino acid decarboxylase activity has better performance in silage with pH 4.87 (CK treatment) than in silage with pH 3.98 (SU treatment) or 4.15 (FA treatment). Metagenomic analysis showed that *Enterobacter cloacae*, *Escherichia coli* and *Citrobacter* sp. carried the genes encoding amino acid decarboxylases and were the potential amine-producing bacteria in alfalfa silage [14]. In addition, our work found that *C. pabulibutyricum* was the predominant undesirable bacterium in the CK silage and had a positive correlation with putrescine, cadaverine and tyramine. It is known that *C. butyricum*, *C. sporogenes*, *C. sphenoides*, *C. tyrobutyricum* and *C. bifermentans* are common *Clostridium* species in silage [46,47]. However, *C. pabulibutyricum* is an infrequently isolated *Clostridium* species and its information has not been well recorded. Kobayashi et al. [48] isolated *C. pabulibutyricum* from high-moisture grass silage and systematically characterized it as a novel species. In addition, Shinha and Hadi [49] reported that *C. pabulibutyricum* can result in bacteremia of a patient with acquired immune deficiency syndrome. In our present work, although the bacterial diversity and their relative abundance in stylo silage were analyzed, we could not ascertain whether these bacteria tested were active or dead during silage fermentation. Therefore, our forthcoming work will attempt to investigate the active bacteria succession during fermentation of stylo silage by means of the metatranscriptomic technique.

## 5. Conclusions

Putrescine, cadaverine, and tyramine were the main biogenic amines and *Clostridium pabulibutyricum* was the predominant undesirable bacterium in spontaneously fermented stylo silage. *C. pabulibutyricum*, *W. cibaria* and *W. paramesenteroides* were positively related to putrescine, cadaverine and tyramine formation. The application of formic acid or sugar significantly decreased biogenic amine concentrations and *C. pabulibutyricum*, *W. cibaria* and *W. paramesenteroides* abundance in silage. These findings lay the foundation for further elucidating the microbial mechanism underlying the main biogenic amine formation during fermentation of stylo silage.

## Figures and Tables

**Table 1:** Chemical characteristics of stylo prior to ensiling (n = 4).

Item ^1^	Fresh Stylo	Standard Deviation
DM, g/kg fresh weight	248	10.08
WSC, g/kg DM	21.5	0.64
pH	5.47	0.05
CP, g/kg DM	132	0.19
NDF, g/kg DM	595	4.94
ADF, g/kg DM	445	8.31
TN, g/kg DM	21.1	0.03
NPN, g/kg TN	125	10.28
Peptide-N, g/kg TN	106	8.49
FAA-N, g/kg TN	16.3	5.46
Ammonia-N, g/kg TN	3.00	1.37

^1^ DM = dry matter; WSC = water-soluble carbohydrates; CP = crude protein; NDF = neutral detergent fiber; ADF = acid detergent fiber; TN = total nitrogen; NPN = non-protein nitrogen; Peptide-N = peptide nitrogen; FAA-N = free amino acid nitrogen; Ammonia-N = ammonia nitrogen.

**Table 2:** Fermentation characteristics of stylo silage (n = 4).

Item ^1^	Treatment ^2^			SEM ^3^	*p*-Value
	CK	FA	SU		
pH	4.87 ^a^	4.15 ^b^	3.98 ^c^	0.14	<0.0001
LA, g/kg DM	12.8 ^b^	0.99 ^c^	57.0 ^a^	8.52	<0.0001
AA, g/kg DM	14.5 ^b^	43.8 ^a^	11.7 ^c^	5.15	<0.0001
LA to AA	0.89 ^b^	0.02 ^c^	4.88 ^a^	0.75	<0.0001
PA, g/kg DM	5.67 ^b^	31.4 ^a^	0.00 ^c^	4.85	<0.0001
BA, g/kg DM	16.1 ^a^	0.00 ^b^	0.00 ^b^	2.73	<0.0001
Ammonia-N, g/kg TN	80.4 ^a^	6.80 ^c^	33.5 ^b^	4.19	<0.0001
V-score	59.5 ^c^	90.0 ^b^	99.3 ^a^	6.06	<0.0001
DM, g/kg fresh weight	243 ^c^	259 ^a^	249 ^b^	2.59	0.0018
WSC, g/kg DM	10.1 ^c^	23.5 ^a^	14.3 ^b^	1.98	<0.0001
CP, g/kg DM	129	130	125	1.82	0.4792
NDF, g/kg DM	578	561	548	9.27	0.459
ADF, g/kg DM	450	419	415	7.36	0.0968
TN, g/kg DM	20.6	20.9	19.9	0.31	0.4663
NPN, g/kg TN	624 ^a^	329 ^b^	374 ^b^	17.75	<0.0001
Peptide-N, g/kg TN	457 ^a^	291 ^b^	271 ^b^	10.58	0.002
FAA-N, g/kg TN	86.6 ^a^	31.0 ^c^	69.2 ^b^	6.75	0.002

^1^ LA = lactic acid; AA = acetic acid; PA = propionic acid; BA = butyric acid; DM = dry matter; Ammonia-N = ammonia nitrogen; TN = total nitrogen; WSC = water-soluble carbohydrates; CP = crude protein; NDF = neutral detergent fiber; ADF = acid detergent fiber; NPN = non-protein nitrogen; Peptide-N = peptide nitrogen; FAA-N = free amino acid nitrogen. ^2^ CK = control; FA = formic acid; SU = sugar. ^3^ SEM, standard error of the mean; Means with different letters (a–c) in the same row indicate a significant difference (*p* < 0.05).

**Table 3:** Amino acids concentrations (mg/kg dry matter) of fresh and ensiled stylo (n = 3).

Item	Fresh Stylo	Treatment ^1^			SEM ^2^	*p*-Value
		CK	FA	SU		
Essential amino acids						
Lysine	295	421 ^b^	353 ^b^	685 ^a^	55.21	0.0043
Methionine	70.6	357 ^a^	69.8 ^c^	213 ^b^	41.98	<0.0001
Tryptophan	122	456 ^a^	78.5 ^c^	217 ^b^	55.38	<0.0001
Valine	233	1359 ^a^	301 ^c^	833 ^b^	155.06	<0.0001
Phenylalanine	324	1548 ^a^	437 ^c^	1049 ^b^	161.78	<0.0001
Leucine	499	2138 ^a^	613 ^c^	1547 ^b^	226.25	<0.0001
Isoleucine	191	1209 ^a^	251 ^c^	732 ^b^	140.22	<0.0001
Threonine	251	620 ^b^	297 ^c^	854 ^a^	81.79	<0.0001
Non-essential amino acids						
Histidine	63.6	470 ^a^	135 ^c^	359 ^b^	50.1	<0.0001
Serine	379	41.4 ^c^	423 ^b^	845 ^a^	121.26	0.0006
Asparagine	2226	743 ^b^	1756 ^a^	1748 ^a^	174.51	0.0004
Glutamine	1122	999 ^a^	348 ^b^	454 ^b^	102.34	<0.0001
Arginine	641	41.0 ^c^	800 ^a^	250 ^b^	113.6	<0.0001
Glycine	146	1358 ^a^	243 ^c^	765 ^b^	162.32	<0.0001
Aspartic acid	365	1712 ^a^	664 ^b^	1512 ^a^	168.44	0.0008
Glutamic acid	376	796 ^a^	547 ^b^	925 ^a^	59.63	0.0023
Alanine	491	2510 ^a^	556 ^c^	1417 ^b^	290.04	0.0001
Proline	305	1386 ^a^	422 ^c^	823 ^b^	142.45	<0.0001
Cystine	0.00	0.00	0.00	0.00	-	-
Tyrosine	202	1136 ^a^	357 ^c^	969 ^b^	119.79	<0.0001
Ornithine	7.63	276 ^b^	17.9 ^c^	512 ^a^	72.54	<0.0001
γ-Aminobutyric acid	202	1177 ^a^	260 ^c^	714 ^b^	137.01	0.0003
D-α-Aminobutyric acid	3.19	310 ^a^	2.45 ^c^	5.87 ^b^	54.12	0.0015
4-Hydroxy-L-Proline	16.6	38.9 ^a^	18.9 ^c^	24.3 ^b^	3.02	<0.0001
Total amino acid	8530	21,102 ^a^	8951 ^c^	17,452 ^b^	1804.45	0.0001

^1^ CK = control; FA = formic acid; SU = sugar. ^2^ SEM, standard error of the mean; Means with different letters (a–c) in the same row indicate a significant difference (*p* < 0.05).

**Table 4:** Biogenic amines concentrations (mg/kg dry matter) of fresh and ensiled stylo (n = 3).

Item	Fresh Stylo	Treatment ^1^			SEM ^2^	*p*-Value
		CK	FA	SU		
Putrescine	125	321 ^a^	150 ^b^	140 ^b^	29.59	<0.0001
Cadaverine	9.26	384 ^a^	21.4 ^c^	144 ^b^	53.59	<0.0001
Tyramine	8.39	127 ^a^	8.68 ^b^	11.0 ^b^	19.66	<0.0001
Spermidine	39.4	16.7 ^c^	35.9 ^a^	28.1 ^b^	2.86	0.0001
Spermine	14.0	4.06 ^c^	6.87 ^a^	5.48 ^b^	0.43	0.0017
Tryptamine	1.67	4.88 ^a^	0.48 ^b^	0.29 ^b^	0.75	<0.0001
Phenylethylamine	1.89	4.17 ^a^	1.53 ^c^	2.32 ^b^	0.4	<0.0001
Histamine	0.00	4.15 ^a^	0.19 ^b^	0.00 ^b^	0.73	0.0026
Total biogenic amine	199	866 ^a^	225 ^c^	331 ^b^	98.03	<0.0001

^1^ CK = control; FA = formic acid; SU = sugar. ^2^ SEM, standard error of the mean; Means with different letters (a–c) in the same row indicate a significant difference (*p* < 0.05).

**Table 5:** Relative abundance (%) of the top six bacteria in stylo silage (n = 3).

Item	Treatment ^1^			SEM ^2^	*p*-Value
	CK	FA	SU		
*Lactiplantibacillus plantarum*	16.3 ^b^	4.17 ^c^	90.6 ^a^	13.6	<0.0001
*Hungatella xylanolytica*	0.00 ^b^	73.6 ^a^	0.01 ^b^	12.57	0.0001
*Clostridium pabulibutyricum*	31.2 ^a^	0.24 ^b^	0.00 ^b^	5.4	0.0005
*Weissella cibaria*	24.1 ^a^	0.50 ^b^	1.60 ^b^	3.88	<0.0001
*Weissella paramesenteroides*	11.8 ^a^	0.31 ^b^	0.51 ^b^	1.91	<0.0001
*Enterococcus* sp. CR Ec1	0.52	7.84	0.00	1.88	0.1636

^1^ CK = control; FA = formic acid; SU = sugar. ^2^ SEM, standard error of the mean; Means with different letters (a–c) in the same row indicate a significant difference (*p* < 0.05).

## Data Availability

All of the sequence data of this study were deposited in the NCBI Sequence Read Archive (SRA) database under the accession number PRJNA1043808.

## Supplementary Materials

The following supporting information can be downloaded at https://www.mdpi.com/article/10.3390/ani14162397/s1. Table S1: V-score evaluation for silage.
