# Supplementary material for: Fermentation Parameters, Amino Acids Profile, Biogenic Amines Formation, and Bacterial Community of Ensiled Stylo Treated with Formic Acid or Sugar
Source: Animals (Basel). 2024 Aug 18;14(16):2397. doi: 10.3390/ani14162397 (PMC11350780; doi:10.3390/ani14162397)
Supplement: Supplementary file 1 [file animals-14-02397-s001.zip › animals-3137725-supplementary.pdf]

Table S1 V-Score evaluation for fresh silage (percent of fresh weight) (Association of Self-supply Feed Evaluation, 2001)

Ammonia nitrogen/total nitrogen (%)		Acetic acid + propionic acid (%)		Butyric acid (%)		V-Score
X_N	Y_N	X_A	Y_A	X_B	Y_B	
≤5	$Y_N=50$	≤0.2	$Y_A=10$	0～0.5	$Y_B=40-80X_B$	
5～10	$Y_N=60-2X_N$	0.2～1.5	$Y_A=(150-100X_A)/13$	0.5＜	0	$Y=Y_N+Y_A+Y_B$
10～20	$Y_N=80-4X_N$	1.5＜	$Y_A=0$			
20＜	$Y_N=0$					
